# Lumbosacral Defects in a 16th–18th-Century Joseon Dynasty Skeletal Series from Korea

**DOI:** 10.1155/2018/7406797

**Published:** 2018-06-27

**Authors:** Yi-Suk Kim, Hankyu Kim, Jong Ha Hong, Hye-Jin Lee, Myeung Ju Kim, Dong Hoon Shin

**Affiliations:** ^1^Catholic Institute for Applied Anatomy, Department of Anatomy, College of Medicine, The Catholic University of Korea, No. 222, Banpo-daero, Seocho-Gu, Seoul 06591, Republic of Korea; ^2^Bioanthropology and Paleopathology Lab, Department of Anatomy, Seoul National University College of Medicine, No. 103, Daehak-ro, Jongno-gu, Seoul 03080, Republic of Korea; ^3^Department of Anatomy, Dankook University College of Medicine, No. 119, Dandae-ro, Dongnam-gu, Cheonan-si, Republic of Korea

## Abstract

Paleopathological evidence for congenital and degenerative disorders of the lumbosacral vertebrae is informative about ancient individual lifeways and physical conditions. However, very few studies have focused on the paleopathology of the lumbosacral vertebrae in ancient skeletal series from East Asia. One reason for the lack of studies is that skeletal samples from East Asia are typically insufficient in size to represent populations for comparative studies within the continent. Here, we present the first comprehensive analysis of lumbosacral defects in an East Asian human skeletal sample, examining occurrences of spina bifida occulta (SBO), lumbosacral transitional vertebrae (LSTV), and spondylolysis in remains from Joseon tombs dating to the 16–18th centuries in Korea. In this study, we present an alternative methodology for understanding activities of daily life among ancient Koreans through paleopathological analysis.

## 1. Introduction

Ancient human skeletal remains, when found in well-preserved condition, allow inferences about the etiology of pathology and make it possible to estimate health conditions in past communities [1]. Paleopathological evidence for congenital and degenerative disorders of the lumbosacral vertebrae is informative about ancient individual lifeways and physical conditions [2]. Pathological findings of the lumbosacral vertebrae, such as spina bifida occulta (SBO), lumbosacral transitional vertebrae (LSTV), and spondylolysis, have been observed in archaic hominins.

SBO is a congenital defect of the vertebral laminae associated with neural tube defects (NTD), or the failure of neural tube closure during embryonic development [3]. In SBO, the lamina have partial defects and the spinal cord is not involved. Both genetic and environmental factors such as folic acid deficiency during pregnancy affect the pathogenesis of NTD [3]. SBO may be located at any level of the neural tube [4].

LSTV is a common developmental malformation in the area of lumbosacral transition [5, 6]. The prevalence of LSTV is reported to vary from 3.3% to 35.6% [7–9]. The most common type of LSTV is the sacralization of L5, which is caused by changes in the shape and size of the L5 processus costalis articulating with the upper edge of S1. The next most common transitional malformations are changes in the number of sacral vertebrae, which may be caused by the lumbarization of S1, by sacralization of Co1, or by the presence of a sixth sacral vertebra [5, 6]. Clinically, LSTV is associated with lower back pain (Bertolotti syndrome) [6, 10]. Degenerative changes in the intervertebral discs may cause lower back pain due to increased flexibility above the transitional vertebrae [6]. Additionally, LSTV significantly impacts the anatomy of surrounding structures in ways that are critical for spinal surgeons to recognize [11, 12].

Spondylolysis is a bony defect in the pars interarticularis of the human vertebrae [13]. This defect is associated with the unique lordotic posture of humans, an adaptation to upright walking [14, 15]. Spondylolytic defects are typically caused by congenital weaknesses or stress fractures [16]. Therefore, the majority of spondylolysis cases involve the lumbar spine, usually L5, and appear bilaterally. Males are affected more often than females because they often engage in more strenuous activities [14, 16, 17].

Lumbosacral defects have been reported in prehistoric and historic skeletal series throughout the world, except Asia [13, 18–22]. Existing reports of lumbosacral defects in Asia are limited to discussions of clinical epidemiology that focus on patient symptoms and complicated analyses of medical images [15, 23–27].

In this study, we present a comprehensive analysis of lumbosacral defects documented in human skeletal remains from Joseon tombs of the 16–18th centuries in Korea. Our data will facilitate a better understanding of the paleopathological prevalence of lumbosacral defects in past Asian peoples. We performed archaeological and anthropological analyses of the Joseon dynasty skeletal series and compare our results to those of previous reports in other countries during similar eras.

## 2. Materials and Methods

We examined a large series of human skeletons (n = 198) making up the Joseon Dynasty Human Sample Collection (JDHSC), which is maintained at Seoul National University College of Medicine, Korea. Most of the skeletons were collected from graves attributed to the Joseon dynasty. Korean archaeologists estimate that these Joseon graves date to the 16th–18th centuries. The Institutional Review Board (IRB) of Seoul National University Hospital confirmed that this study was exempt from board review (IRB no. 2017-001). We followed the guidelines of the Vermillion Accord on Human Remains, World Archaeological Congress [28].

Sex determinations were made on the basis of pelvic morphology, including examinations of the greater sciatic notch, preauricular sulcus, ischiopubic ramus, subpubic angle, subpubic concavity, and ventral arc [29, 30]. Ancillary indicators used for sex determination included characters of the skull, specifically the nuchal crest, the mastoid process, the supraorbital margin, glabella, and the mental eminence [31, 32].

Incomplete midline closure of the lumbosacral vertebrae indicated SBO, regardless of mesenchymal, osseous, or neural tissue origin during embryological development. Following previous definitions drawn from the literature, SBO at S3, S4, and S5 was considered within the limits of normal variation for the sacral hiatus [33]. LSTV was identified only if all of the lumbosacral vertebrae were present. After recording the number of lumbar vertebrae and sacral segments, the conversion of L5 or L6 into S1 was defined as sacralization and the reverse as lumbarization. The presence of an extra L6 with 5 sacral segments was defined as a form of LSTV in this study, including the lumbarization of S1 in cases of six sacral vertebrae and the sacralization of Co1 [5, 6]. Defects of the pars interarticularis of the vertebrae were examined in cases of spondylolysis [15]. To prevent bias, we excluded cases of fresh spondylolysis that were difficult to differentiate from postmortem fractures of the pedicle.

We processed the data using descriptive statistics. Differences according to sex and age were evaluated using Fisher's exact test with a significance level of 5%, using R version 3.4.0 (R Foundation for Statistical Computing, Vienna, Austria).

## 3. Results

**Figure 1:**
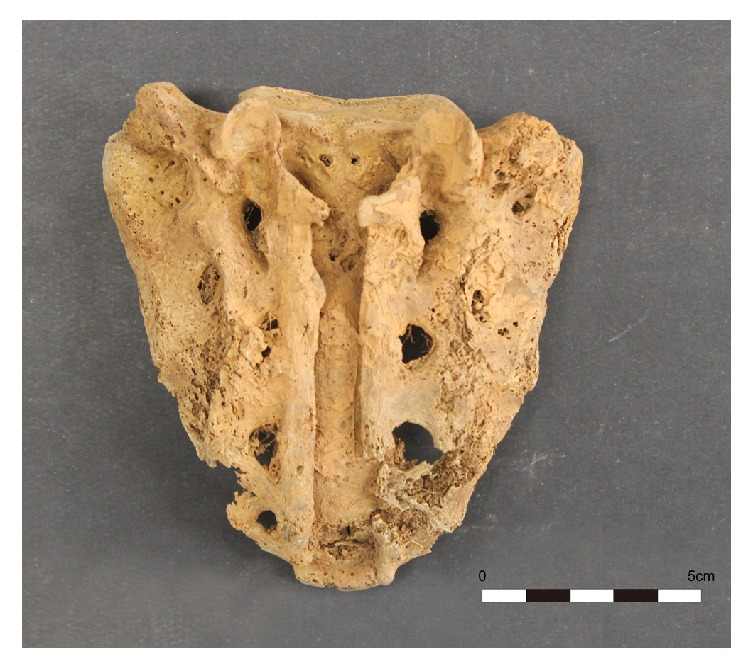
Sacral spina bifida (S1–S5) occulta in a male (Case no. 190).

The skeletal sample included 81 males, 68 females, and 49 indeterminates. Lumbosacral data are presented in Tables 1–4. The most frequent lumbosacral defect was SBO, followed by sacralization, and lumbarization or spondylolysis (Table 1). In cases of SBO, S1 was the most frequent defect site (8 of 198, 4.0%), followed by S2 (4 of 198, 2.0%) and L5 (2 of 198, 1.0%). Four male cases (1 case at S3, 3 cases at S4) and 3 female cases (2 cases at S3, 1 case at S4) were not regarded as SBO, but as congruent with normal variation of sacral hiatus. SBO was more frequently observed in males, especially at S1, than in females (Table 2). However, the difference between sexes was not significant by Fisher's exact test (p = 0.143). In one male case (no. 190), the SBO defect involved all segments of the sacrum as seen in Figure 1.

**Figure 2:**
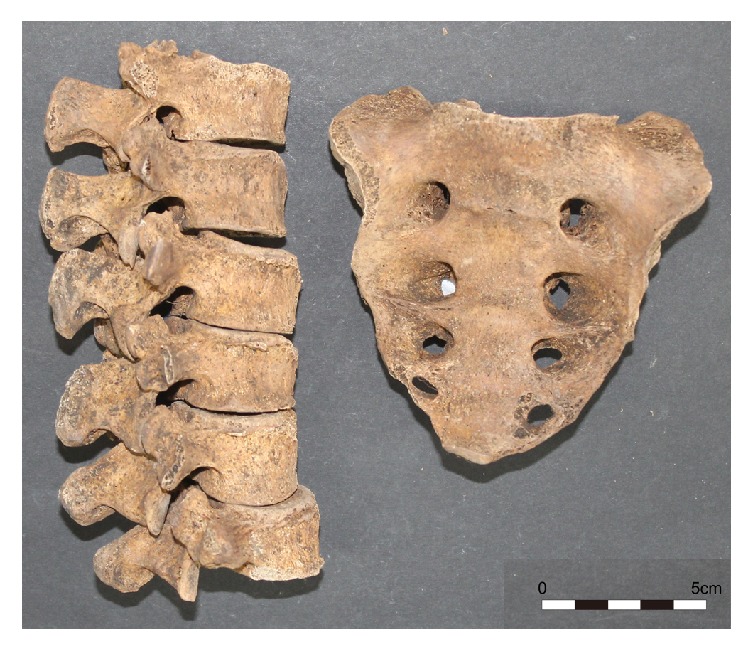
Extra (sixth) lumbar vertebra (left) with normal sacrum consisting of 5 sacral segments (right) in a female (Case no. 113).

The prevalences of total LSTV were approximately 14% in males and 4% in females (Table 3). Males were more likely to exhibit sacralization of L5 and L6 than females (p = 0.022, data not shown) although the difference in cases of total LSTV (sacralization plus lumbarization) between sexes was not significant (p = 0.088). In the same skeletal series, we also note that two cases, one female (no. 113) and one male (no. 162), exhibited extra L6 alongside 5 sacral segments (Table 3, Figure 2). These cases were considered to represent the lumbarization of S1 among the six sacral vertebrae that were present.

**Figure 3:**
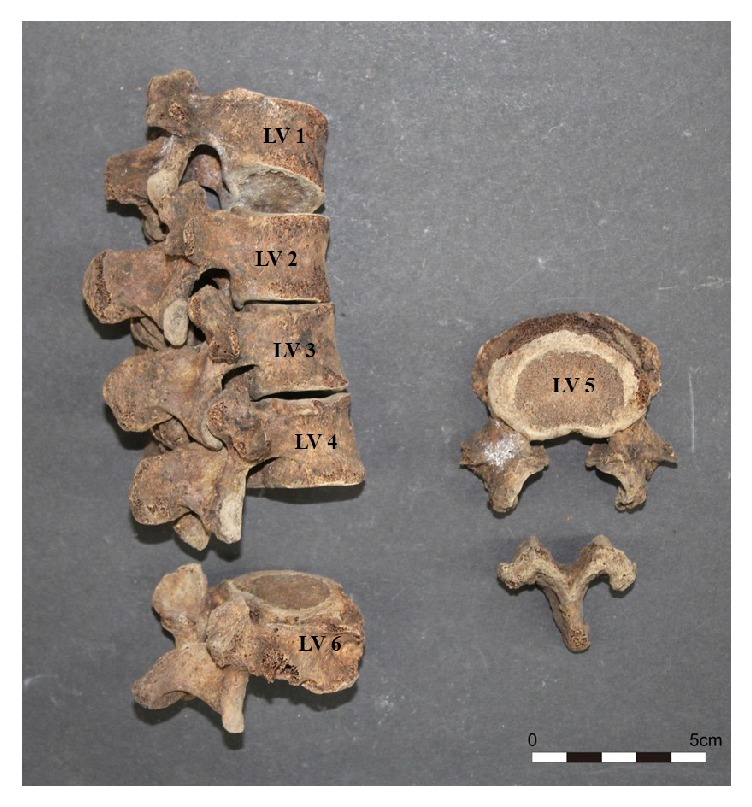
Spondylolysis of L5 with six lumbar vertebrae in a specimen of unknown sex and age (Case no. 289).

The prevalence of spondylolysis according to sex is shown in Table 4. Overall, the most frequent defect site was L5 (4 of total 198, 2.0%) but no defects were found in the sacrum. There were no significant differences between sexes (Table 4). Among the sex-indeterminate skeletons, we identified a unique case (no. 289) with an extra L6 and spondylolysis at L5 (Figure 3).

## 4. Discussion

NTD, including SBO, are some of the most common types of congenital malformations, occurring at 21 to 28 days after conception [34]. The general prevalence of NTD is estimated to be 0.51–4.2 per 1000 live births, stillbirths, and pregnancy terminations in the modern period [35, 36]. The birth prevalence of spina bifida may differ considerably depending on geographical and population origin [37].

SBO is the most common developmental defect of the vertebral column observed in historical skeletal series, occurring frequently at the lumbosacral border, with reported incidences of 5.2% to 26.0% [13]. In our study of Joseon skeletons, we also found that SBO was the most commonly observed pathology in the lumbosacral vertebrae. There are significant temporal and geographic influences on maternal nutrition, including folic acid supplementation, affecting the etiology of spina bifida [34]. When we compared data for the Joseon skeletal series to the results of previous studies examining different countries and different historical periods, SBO tended to have higher prevalence in the Joseon sample than in most historical populations, excepting medieval Slovakia (Table 5). The prevalence of SBO in Slovakian skeletal series [13] may have been higher than the prevalence of SBO in Korea during the Joseon dynasty, because the majority of lower sacral segment defects (S4-S5, S3–S5) were regarded as SBO, which was in turn defined as being with the range of normal variation for the sacral hiatus. Taken together, these observations indicate that the prevalence of SBO among the Joseon people is one of the highest ever observed among ancient skeletal series. Neurological defects, particularly spina bifida, have actually revealed a significant decrease over time due to the supplementation of food with fortified folic acid worldwide [38]. Therefore, the high prevalence of SBO in our Joseon dynasty sample might have been caused by maternal folate insufficiencies that were more serious than in other populations.

The lumbosacral region is the most frequent site of border shifting [13]. Abnormal cranial shifting leads to the sacralization of the fifth lumbar vertebra, while caudal shifting leads to lumbarization of the first sacral vertebra. In both cases, the defect may be complete or incomplete, unilateral or bilateral, and symmetrical or asymmetrical. According to previous studies, the general prevalence of LSTV ranges from 4% to 35.6% in different populations, and males are more frequently affected than females [7–9]. Compared with samples from different countries but of similar era, differences of prevalence of LSTV between Joseon-Korea and other medieval countries prove to be minor, with a slight tendency for the prevalence of LSTV to be higher in males than in females, a pattern that holds true throughout a variety of countries (Table 5).

In the literature, spondylolysis is described as ossification union failure or the fracture of the pars interarticularis of the vertebra, resulting in separation of the vertebra into two parts. This separation occurs most commonly in S1 and L3–L5 [13]. The prevalence of lumbar spondylolysis is estimated to be 5% in the general population but can be as high as 63% in people who participate in certain sporting activities and also varies between populations [14, 16, 39]. For example, 13% of a sample of Canadian Inuits exhibited spondylolysis, while the prevalence was 18.5% in a sample of Archaic Indians [13]. Approximately 20.7% of Japanese athletes are affected by spondylolysis [40]. Lumbar spondylolysis is thought to result from stress fractures of the pars interarticularis that occur frequently in athletes. The Joseon sample examined in this study was assumed to represent individuals only infrequently engaged in mechanically demanding activities, with a total incidence of spondylolysis (3.0%) lower than that of any other known ancient skeletal series (Table 5). The prevalence is also low compared to modern Koreans, in which population values range from 5.9% to 9.1% [14, 41]. The low frequency of spondylolysis observed in the Joseon sample suggests a lack of heavy physical labor in this population [29]. As the individuals in the sample primarily represent the upper and ruling classes of the Joseon kingdom [42], the relatively low prevalence of spondylolysis we observed could reflect socioeconomic differences. However, comparisons of disease in different skeletal series must be approached carefully if research methods used have not been standardized [34].

## 5. Conclusion

Very few studies have focused on the paleopathology of lumbosacral vertebrae in ancient skeletal series from East Asia. One reason for the lack of studies is that skeletal samples from East Asia are typically insufficient in size to represent populations for comparative studies within the continent. This paleopathological study of lumbosacral pathologies in a large sample from the Joseon era is thus significant. Our results suggest that chronic scarcities of folic acid were prevalent during the Joseon dynasty, as evidenced by the high incidence of SBO, similar to other medieval samples. In contrast, the relatively low prevalence of spondylolysis may reflect a lack of strenuous physical labor among the ruling classes of Joseon society. Differences in the prevalence of LSTV between Joseon people in Korea and medieval samples from other countries are minor, indicating that genetic causes are unlikely. This study contributes to our understanding of daily life among ancient Koreans through paleopathology.

## Figures and Tables

**Table 1:** Frequencies of lumbosacral defects observed in this study (n=198).

	Number of findings (%)
Spina bifida occulta	15 (7.6)
Sacralization	10 (5.1)
Lumbarization	6 (3.0)
Spondylolysis	6 (3.0)

**Table 2:** Descriptive statistics of spina bifida occulta (SBO) in Joseon males and females.

	Number of SBO occurrences/total (%)			P value*∗*
	Males	Females	Indeterminate	
Spina bifida at L5	1/81 (1.2%)	1/68 (1.5%)	0/49 (0.0%)	1.000
Spina bifida at S1	6/81 (7.4%)	1/68 (1.5%)	1/49 (2.0%)	0.126
Spina bifida at S2	2/81 (2.5%)	1/68 (1.5%)	1/49 (2.0%)	1.000
Spina bifida at S1–S5	1/81 (1.2%)	0/68 (0.0%)	0/49 (0.0%)	1.000
Total	10/81 (12.3%)	3/68 (4.4%)	2/49 (4.1%)	0.143

*∗* probability value of Fisher's exact test between the sexes.

**Table 3:** Descriptive statistics of lumbosacral transitional vertebrae (LSTV) in Joseon males and females.

	Number of LSTV occurrences/Total (%)			P value*∗*
	Males	Females	Indeterminate	
Sacralization at L5	7/81 (8.6%)	1/68 (1.5%)	0/49 (0.0%)	0.071
Sacralization at L6	2/81 (2.5%)	0/68 (0.0%)	0/49 (0.0%)	0.501
Lumbarization at S1	1/81 (1.2%)	1/68 (1.5%)	2/49 (4.1%)	1.000
Extra L6 with 5 sacral segments	1/81 (1.2%)	1/68 (1.5%)	0/49 (0.0%)	1.000
Total	11/81 (13.6%)	3/68 (4.4%)	2/49 (4.1%)	0.088

*∗* probability value of Fisher's exact test between the sexes.

**Table 4:** Descriptive statistics of spondylolysis in Joseon males and females.

	Number of spondylolysis occurrences / total (%)			P value*∗*
	Males	Females	Indeterminate	
Spondylolysis at L2	1/81 (1.2%)	0/68 (0.0%)	0/49 (0.0%)	1.000
Spondylolysis at L4	1/81 (1.2%)	0/68 (0.0%)	0/49 (0.0%)	1.000
Spondylolysis at L5	1/81 (1.2%)	2/68 (2.9%)	1/49 (2.0%)	0.592
Total	3/81 (3.7%)	2/68 (2.9%)	1/49 (2.0%)	1.000

*∗*Probability value of Fisher's exact test between the sexes.

**Table 5:** Prevalence of lumbosacral malformations in different samples during similar eras.

Country	Century	Prevalence (%)											
		Spina bifida occulta			Sacralization			Lumbarization			Spondylolysis		
		Male	Female	Total	Male	Female	Total	Male	Female	Total	Male	Female	Total
Joseon Korea (This study)	16-18th	12.3	4.4	7.6	9.9	1.4	4.5	4.9	2.9	4.0	3.7	2.9	3.0
British **[18]**	10-19th	-	-	-	-	-	-	-	-	7.0	14.5	8.9	11.9
Bulgaria **[19]**	9-15th	6.6	0	3.8	-	-	2.9	-	-	-	-	-	-
France **[21]**	5-11th	-	-	-	-	-	3.3	-	-	3.3	-	-	10
Hungary **[22]**	10-17th	7.1	3.3	4.2	2.7	0.9	1.4	3.5	-	1.4	-	-	-
Romania **[20]**	15-19th	5.2	1.9	3.8	-	-	-	-	-	-	-	-	-
Slovakia **[13]**	11-12th	26.0	19.0	22.0	15.0	2.0	8.0	2.0	2.0	2.0	8.0	8.0	7.0

## Data Availability

The data used to support the findings of this study are available from the corresponding author upon request.
